# Improved neurologically favorable survival after OHCA is associated with increased pre-hospital advanced airway management at the prefecture level in Japan

**DOI:** 10.1038/s41598-022-25124-2

**Published:** 2022-11-28

**Authors:** Atsunori Onoe, Kentaro Kajino, Mohamud R. Daya, Fumiko Nakamura, Mari Nakajima, Masanobu Kishimoto, Kazuhito Sakuramoto, Takashi Muroya, Hitoshi Ikegawa, Marcus Eng Hock Ong, Yasuyuki Kuwagata

**Affiliations:** 1 Department of Emergency and Critical Care Medicine, Kansai Medical University, Shinmachi 2-5-1, Hirakata, Osaka 573-1010 Japan; 2 Department of Emergency Medicine, Oregon Health and Science University, Portland, OR USA; 3 Department of Emergency Medicine, Singapore General Hospital, Singapore, Singapore; 4 Health Services and Systems Research, Duke-NUS Medical School, Singapore, Singapore

**Keywords:** Cardiology, Medical research

## Abstract

Out-of-hospital cardiac arrest (OHCA) has high incidence and mortality. The survival benefit of pre-hospital advanced airway management (AAM) for OHCA remains controversial. In Japan, pre-hospital AAM are performed for OHCA by emergency medical services (EMS), however the relationship between resuscitation outcomes and AAM at the prefecture level has not been evaluated. The purpose of this study was to describe the association between AAM and neurologically favorable survival (cerebral performance category (CPC) ≦2) at prefecture level. This was a retrospective, population-based study of adult OHCA patients (≧ 18) from January 1, 2014 to December 31, 2017 in Japan. We excluded patients with EMS witnessed arrests. We also only included patients that had care provided by an ELST with the ability to provided AAM and excluded cases that involved prehospital care delivered by a physician. We categorized OHCA into four quartiles (four group: G1–G4) based on frequency of pre-hospital AAM approach rate by prefecture, which is the smallest geographical classification unit, and evaluated the relationship between frequency of pre-hospital AAM approach rates and CPC ≦ 2 for each quartile. Multivariable logistic regression was used to assess effectiveness of AAM on neurologically favorable survival. Among 493,577 OHCA cases, 403,707 matched our inclusion criteria. The number of CPC ≦ 2 survivors increased from G1 to G4 (*p* for trend < 0.001). In the adjusted multivariable regression, higher frequency of pre-hospital AAM approach was associated with CPC ≦ 2 (*p* < 0.001). High prefecture frequency of pre-hospital AAM approach was associated with neurologically favorable survival (CPC ≦ 2) in OHCA.

## Introduction

Out-of-hospital cardiac arrest (OHCA) is a major public health concern due to its high incidence and mortality^1–3^. Basic life support (BLS), which consists of early cardiopulmonary resuscitation (CPR) and timely defibrillation, improves outcomes in patients with OHCA^4–6^.

Early activation of emergency medical services (EMS) is one of the key components in the OHCA chain of survival. The provision of oxygenation and ventilation of the lungs via bag-valve-mask (BVM) devices, supraglottic airway placement (SGA), or endotracheal intubation (ETI) are important additional resuscitation skills delivered by EMS personnel^7^. Recent studies have raised questions about the survival benefits of advanced airway management (AAM) with the use of SGA or ETI compared to BVM ventilation in OHCA patients^8–15^. However, there have not been any definitive studies evaluating the association between AAM strategies and patient centered outcomes such as survival with good neurological status.

A better understanding of the relationship between factors influencing prehospital Return of Spontaneous Circulation (ROSC) and neurologically favorable survival following OHCA is important for countries with a growing aging population such as Japan. In Japan, there are differences in the frequency of pre-hospital AAM approach by EMS personal for OHCA across prefectures (regions), and resuscitation outcome differences based on frequency of pre-hospital AAM approach has not been well studied.

The purpose of this study was to evaluate the association between frequency of pre-hospital AAM approach at a prefecture level, which is the smallest classification unit, and good functional survival.

## Methods

### Study design, setting, population

This was a retrospective cohort study examining data from January 1, 2014 to December 31, 2017 reported to the All-Japan Utstein Registry database. This study will calculate frequency of pre-hospital AAM approach rates by prefecture and compare and contrast it by prefecture. We included OHCA patients who were 18 years of age or older. The All-Japan Utstein Registry of the Fire and Disaster Management Agency (FDMA) is a prospective, population based, nationwide registry of OHCA launched in January 2005, which records resuscitation related data according to the international Utstein-style reporting system^16^. Details of the registry have been described previously^17^. Cardiac arrest was defined as the cessation of cardiac mechanical activity, as confirmed by the absence of signs of circulation^16^. Each EMS authority submitted anonymized data to the registry.

### The EMS system in Japan

**Figure 1:**
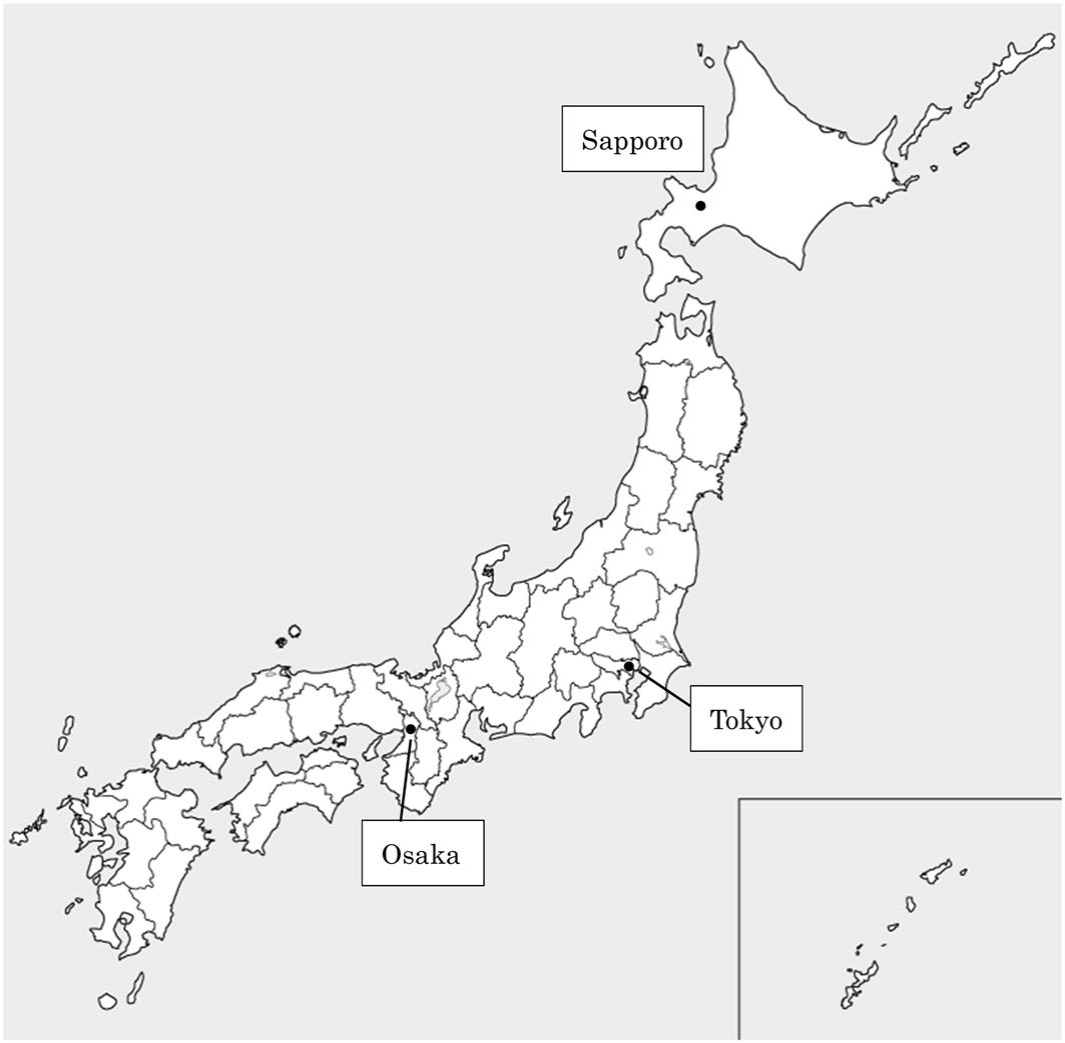
Map of Japan. In the present study, the 47 prefectures of Japan.

Japan has a total land area of 377,976 km^2^ and is divided into 47 prefectures as a large area which has its own local government like states (Fig. 1). The population is about 126 million, and the population density is 338.22 persons per square meter. Prehospital life support is provided 24 h a day through a Fire-based EMS system. Among EMS personnel, specially trained emergency care providers known as emergency life-saving technicians (ELSTs) are authorized to use automated external defibrillators. Since the 1991 enactment of the ELST Law, most ambulances in Japan are manned by at least one ELST. They resuscitate OHCA patients according to protocols developed by local medical control councils based on recommended guidelines from the Japan Resuscitation Council and International Liaison Committee on Resuscitation. As for prehospital medical control (MC), each prefecture has its own prefectural MC council. MC council and there are 251 regional MC councils based on additional regional characteristics throughout Japan.

In 1991, ELST were also permitted to use SGA devices (laryngeal mask airway, laryngeal tube, and esophageal-tracheal twin lumen airway device) for patients with OHCA under medical control direction^18^. Since 2004, ETI can be performed by specially trained ELSTs who have completed an additional 62 h of training sessions and have performed 30 supervised successful intubations in operating rooms^19^. Each prefectural and regional MC council examines and implements emergency operation protocols independently^20,21^. With support and direction from online medical control, specially trained ELSTs are also permitted to perform other advanced life care including administering adrenaline and performing ETI in patients with OHCA. In Japan, EMS providers are generally not permitted to terminate resuscitation in the field, and all patients who have resuscitation attempted must be transported to the hospital. Resuscitation is not attempted under particular circumstances (e.g. decapitation, incineration, decomposition, rigor mortis, or dependent cyanosis). Although all ELSTs can use SGA devices such as laryngeal tubes or laryngeal masks, only specially trained, certified ELSTs are permitted to perform endotracheal intubation under direction from online medical control. These ELSTs are allowed to perform endotracheal intubation during cardiac arrest and are not permitted to intubate following ROSC. Treatment for cardiac arrest during the study time period were based on the Japanese cardiopulmonary resuscitation guidelines^22^, which are based on the consensus statements of the International Liaison Committee on Resuscitation (ILCOR)^23^.

In Japan, specially trained ELSTs are authorized to perform ETI under medical direction for cardiac arrest patients (lack of pulse and apnea) as follows: 1. The patient meets indications for endotracheal intubation. 2. It is impossible to maintain the patient’s airway with SGA, because of (a) asphyxia due to foreign-body airway obstruction or, (b) in cases in which the medical control doctor judges ETI to be required. 3. Patients are ineligible for endotracheal intubation, if there is: (i) suspected cervical spine injury, (ii) head-tilt difficulty, (iii) trismus, (iv) difficulty with laryngoscope insertion, (v) difficulty with larynx expansion after laryngoscope insertion, (vi) difficulty in visualizing the vocal chords, (vii) prolonged unsuccessful attempts, (viii) the ELST on the scene is not certified to perform endotracheal intubation, and (ix) rapid sequence intubation (paralysis and sedation) is not permitted at any time.

### Selection of participants and ethics

We included patients with complete data who were 18 years of age and above, and excluded EMS witnessed and cases where the witness status was unknown. We also only included patients that had care provided by an ELST with the ability to provided AAM and excluded cases that involved prehospital care delivered by a physician at any time. In this study, cases of failed attempts at AAM were classified as cases without AAM. The study protocol have been performed in accordance with the Declaration of Helsinki, and was approved by the Ethics Committee of Kansai Medical University. The requirement for individual informed consent was waived per the Personal Information Protection Law and National Research Ethics Guideline in Japan.

### Data collection and quality control

Data was collected with the use of a form based on the Utstein guidelines for reporting OHCA, and included details on age, gender, witness status, initial cardiac rhythm, time course of resuscitation, bystander-initiated CPR, AAM use, adrenaline administration, as well as pre-hospital return of spontaneous circulation (ROSC), one-month survival, and neurological status one month after the event. The time course of EMS activity included both response time (from emergency call to EMS arrival on scene) and total prehospital time (from emergency call to EMS hospital arrival). All survivors were evaluated at one month after the event for neurological function. The data form was filled out by the EMS personnel in cooperation with the physicians in charge of the patients, and the data were integrated into the registry all data was logic-checked by the computer system and were confirmed by the implementation working group. If a data form was incomplete, the FDMA would return it to the respective fire station for completion and follow up on any missing data^24^.

### Outcomes

Neurological outcome was clinically determined by the physician caring for survivors one month after successful resuscitation, using the cerebral performance category (CPC) scale: category 1, good cerebral performance; category 2, moderate cerebral disability; category 3, severe cerebral disability; category 4, coma or vegetative state; and category 5, death. The primary outcome measure was neurologically favorable one-month survival, defined as CPC category 1 or 2. Secondary outcome was survival at one month and ROSC.

### Statistical analysis

AAM proportional use for OHCA cases was calculated for the included cases by prefecture and the prefectures were classified into four groups based on quartiles according to frequency of pre-hospital AAM approach rates.

Patient and EMS characteristics as well as outcomes were evaluated for each quartile group. As a secondary analysis, we evaluated cases where AAM was performed within each group. Patient characteristics were compared using chi-square test for categorical variables and one-way analysis of variance for ordinal variables. EMS characteristics such as procedures and care time intervals were tested with univariable regression models for categorical variables and linear tests for numerical variables. Logistic regression for CPC 1 or 2 was used to adjust for confounding factors. Odds ratios (ORs) and their 95% confidence intervals (CIs) were calculated, controlling for age, gender, witnessed status, bystander CPR, presenting initial rhythm (shockable or non-shockable), prehospital defibrillation at any time, response time and total prehospital time. All statistical analyses were performed using SPSS statistical package ver. 23.0 J (SPSS, Inc., Chicago, IL). All tests were 2-tailed, and *P* values of < 0.05 were considered statistically significant.

### Ethics approval and consent to participate

All participating physicians consented to participate.

## Results

**Figure 2:**
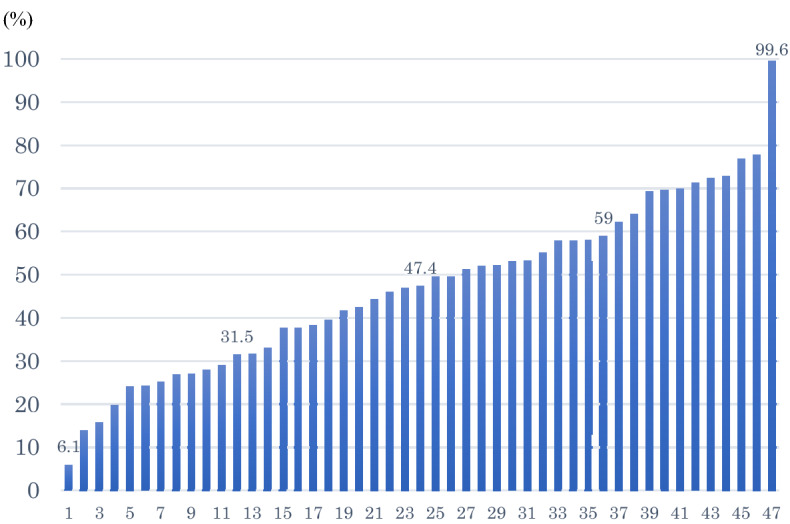
The frequency of pre-hospital advanced airway management approach for cardiac arrest cases in the prefecture.

**Figure 3:**
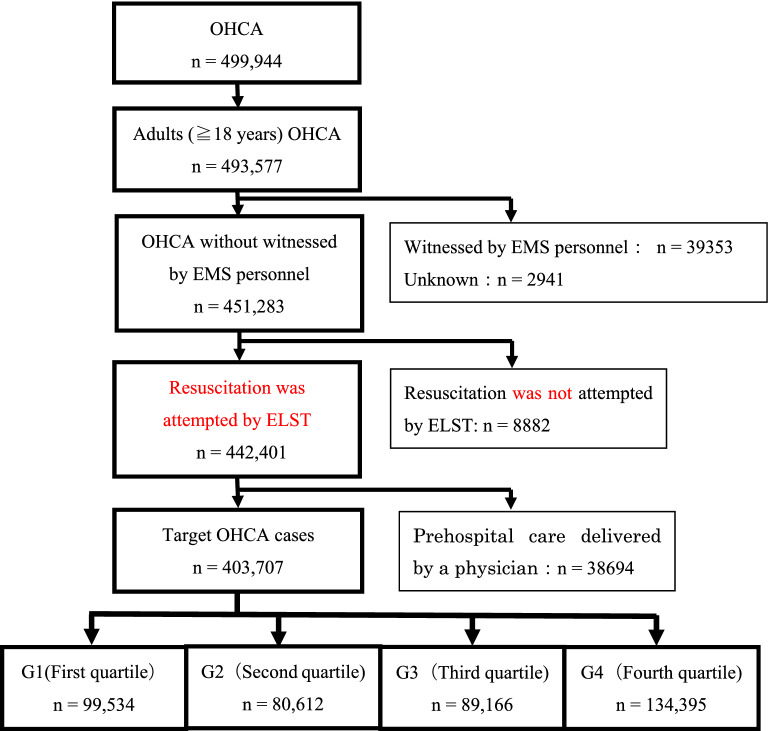
Flow of out-of-hospital cardiac arrest patients from January 1, 2014 to December 31, 2017 in the All-Japan Utstein Registry. *Cases grouped by the frequency of pre-hospital Advanced Airway Management approach by prefecture (Quartile G1 to G4).

During the study period from January 1, 2014 to December 31, 2017, 493,577 OHCA cases, ≥ 18 years of age were reported in the All-Japan Utstein Registry. The number of cases that met the study inclusion criteria was 403,707 cases. The frequency of pre-hospital AAM approach of each prefecture was calculated, and the mean proportion was 47.4%, the minimum was 6.1%, and the maximum was 99.6% (Fig. 2). The quartile of the AAM implementation proportion was 31.5% for the 25th percentile, 47.4% for the 50th percentile, and 59% for the 75th percentile. The 403,707 cases were then classified as follows according to the quartiles according to AAM use; the first quartile (G1, < 31.5%): 99,534 cases, 2nd quartile (G2, 31.5–47.4%): 80,612 cases, 3rd quartile (G3, 47.4–59%): 89,166 cases, 4th quartile (G4, ≧59%): 134,395 cases (Fig. 3).

The characteristics of each group (G1-G4) were as follows. The mean age was G1: 75 ± 16 years, G2: 77 ± 15 years, G3: 76 ± 15 years, G4: 76 ± 16 years. AAM use for G1 was: 18,090 cases 18.2%, G2: 28,167 cases 34.9%, G3: 41,878 cases 47.0%, G4: 75,439 cases 56.1%. The number of ETI and SGA performed in groups G1 to G4 were as follows; (G1: ETI 3327 cases, SGA 14,763 cases, G2: ETI 6340 cases, SGA 21,827 cases, G3: ETI 5727 cases, SGA 36,151 cases, G4: ETI 15,075 cases, SGA 60,364 cases). Epinephrine administration tended to be lower in G1 and higher in G4; (G1: 11,479 cases 11.5%, G2: 10,955 cases 13.6%, G3: 17,840 cases 20.0%, G4: 36,503 cases 27.2%) (Table 1). As the frequency of pre-hospital AAM approach increased from G1 to G4, the *p* for trend showed a significant improvement in all outcomes including prehospital ROSC, 1-month survival, and 1-month of patients with a CPC1 or 2 group (Table 1). In multivariate analysis for CPC 1or 2 after adjusting for confounders, the key predictor variables were: Witnessed by bystander (OR 6.050, 95% CI: 5.716–6.404, *p* < 0.001), bystander CPR (OR 2.537, 95% CI: 2.411–2.670, *p* < 0.001), and initial shockable rhythm (OR 6.078, 95% CI 5.748–6.427, *p* < 0.001). Factors associated with poor prognosis included: one year increase in age (OR 0.969, 95% CI 0.968–0.970, *p* < 0.001), AAM implementation (OR 0.225, 95% CI 0.211–0.239, *p* < 0.001), and epinephrine administration (OR 0.302, 95% CI 0.280—0.326, *p* < 0.001) (Table 2). High AAM implementation was associated with better neurological outcome (CPC 1 or 2) (G1 Reference; G2 OR 1.203, 95% CI 1.121–1.285, *p* < 0.001; G3 OR 1.675, 95% CI 1.566–1.792, *p* < 0.001; G4 OR 1.859, 95% CI 1.743–1.982, *p* < 0.001) (Table 2).

**Table 1:** Background of OHCA cases and OHCA survival outcomes grouped by the frequency of pre-hospital Advanced Airway Management approach (Quartile G1 to G4).

	G1	G2	G3	G4	*P* for trend
**Background of OHCA**					
Number of cases	99,534	80,612	89,166	134,395	
Age (y.o), mean (S.D.)	75.6 ± 15.9	76.6 ± 15.2	76.3 ± 15.3	75.6 ± 15.7	0.88
Male, n (%)	56,283 (56.5)	45,461 (56.4)	50,534 (56.7)	75,732 (56.4)	< 0.001
Presumed cardiac causes, n (%)	59,009 (59.3)	47,814 (59.3)	53,290 (59.8)	87,644 (65.2)	< 0.001
Cerebrovascular disease, n (%)	2711 (2.7)	2756 (3.4)	2911 (3.3)	3523 (2.6)	
Respiratory disease, n (%)	7592 (7.6)	7414 (9.2)	7567 (8.5)	11,040 (8.2)	
Malignant tumor, n (%)	3189 (3.2)	2332 (2.9)	2762 (3.1)	3965 (3.0)	
External causes, n (%)	7107 (7.1)	4910 (6.1)	5138 (5.8)	8180 (6.1)	
Other, n (%)	19,926 (20.1)	15,386 (19.1)	17,498 (19.5)	20,043 (14.9)	
Witnessed by bystander, n (%)	37,029 (37.2)	28,405 (35.2)	32,020 (35.9)	48,488 (36.1)	< 0.001
Bystander CPR, n (%)	47,815(48.0)	42,356 (52.5)	50,341 (56.5)	69,456 (51.7)	< 0.001
Initial rhythm VF, n (%)	6144 (6.2)	4821 (6.0)	5553 (6.2)	8546 (6.4)	< 0.001
AAM, n (%)	18,090 (18.2)	28,167 (34.9)	41,878 (47.0)	75,439 (56.1)	< 0.001
ETI, n (%)	3327 (3.3)	6340 (7.9)	5727 (6.4)	15,075 (11.2)	
SGA, n (%)	14,763 (14.9)	21,827 (27.0)	36,151 (40.6)	60,364 (44.9)	
Epinephrine, n (%)	11,479 (11.5)	10,955 (13.6)	17,840 (20.0)	36,503 (27.2)	< 0.001
**EMS care interval (min), mean (S.D.)**					
Call to ambulance arrives on the scene	7.7 ± 3.7	8.6 ± 4.1	8.3 ± 3.6	7.4 ± 3.2	< 0.001
Call to AAM implementation	19.9 ± 7.7	20.6 ± 8.3	18.4 ± 7.3	17.3 ± 6.1	< 0.001
Call to Epinephrine	24.6 ± 7.6	26.9 ± 8.5	24.2 ± 8.4	23.7 ± 7.8	< 0.001
Call to hospital arrival	35.3 ± 12.7	34.2 ± 12.7	32.2 ± 11.9	33.7 ± 12.9	< 0.001
**Survival outcomes of OHCA**					
ROSC in the field, n (%)	8446 (8.5)	5915 (7.3)	8043 (9.0)	13,361 (9.9)	< 0.001
Survival at one month, n (%)	5061 (5.1)	3935 (4.9)	4466 (5.0)	8043 (6.0)	< 0.001
CPC 1 or 2, n (%)	2870 (2.9)	2113 (2.6)	2567 (2.9)	4275 (3.2)	< 0.001

*OHCA* Out-of-hospital cardiac arrest, *CPR* cardiopulmonary resuscitation, *AAM* advanced airway management, *ETI* endotracheal intubation, *SGA* supraglottic airway placement, *EMS* emergency medical service, *ROSC* return of spontaneous circulation, *CPC* the cerebral performance category.

**Table 2:** Multivariate analysis for Survival with CPC1 or 2.

	OR	95% CI		*P*
Age	0.969	0.968	0.970	*P* < 0.001
Male	1.218	1.156	1.284	*P* < 0.001
Presumed cardiac causes	1.626	1.537	1.721	*P* < 0.001
Witnessed by bystander	6.050	5.716	6.404	*P* < 0.001
Bystander CPR	2.537	2.411	2.670	*P* < 0.001
initial shockable rhythm	6.078	5.748	6.427	*P* < 0.001
AAM implementation	0.225	0.211	0.239	*P* < 0.001
Epinephrine administration	0.302	0.280	0.326	*P* < 0.001
Call to ambulance arrives on the scene	0.918	0.910	0.926	*P* < 0.001
Call to hospital arrival	1.012	1.011	1.014	*P* < 0.001
**AAM implementation proportion by prefecture**				
G1 (< 31.5%)	Reference			
G2 (31.5–47.4%)	1.203	1.121	1.285	*P* < 0.001
G3 (47.4–59%)	1.675	1.566	1.792	*P* < 0.001
G4 (≧59%)	1.859	1.743	1.982	*P* < 0.001

*CPC* the cerebral performance category, *OR* odds ratio, *CI* confidence interval, *CPR* cardiopulmonary resuscitation, *AAM* advanced airway management.

In a secondary analysis, we also analyzed only the OHCA cases with AAM implemented separately. The characteristics of the AAM cases in each quartile (G1 to G4) were as follows. The mean age was G1: 76 ± 14 years, G2: 77 ± 14 years, G3: 77 ± 14 years, G4: 76 ± 15 years. The percentages of ETI and SGA in groups G1 to G4 were as follows; (G1: ETI 18.3%, SGA 81.7%, G2: ETI 22.5%, SGA 77.5%, G3: ETI 13.7%, SGA 86.3%, G4: ETI 20.0%, SGA 80.0%). Epinephrine administration tended to be lower in G1 and higher in G4; (G1: 4530 cases 25.0%, G2: 5968 cases 21.2%, G3: 11,753 cases 28.0%, G4: 29,779 cases 39.5%) (Table 3). As AAM use increased from G1 to G4, the *p* for trend showed a significant difference, and improvement in survival was observed in all outcomes including prehospital ROSC, 1-month survival, and 1-month of patients with a CPC1 or 2 group (Table 3). In multivariate analysis for CPC 1or 2 after adjusting for confounders, the key predictor variables were: Witnessed by bystander (OR 3.480, 95% CI: 3.068–3.948, *p* < 0.001), and initial shockable rhythm (OR 12.54, 95% CI 11.10–14.16, *p* < 0.001). Factors associated with poor prognosis included: one year increase in age (OR 0.968, 95% CI 0.968–0.974, *p* < 0.001), and epinephrine administration (OR 0.641, 95% CI 0.575–0.716, *p* < 0.001) (Table 4). High AAM implementation was associated with a better neurological outcome (CPC 1 or 2) (G1 reference; G2 OR 1.158, 95% CI 0.924–1.453, *p* = 0.203; G3 OR 1.717, 95% CI 1.408–2.093, *p* < 0.001; G4 OR 1.723, 95% CI 1.432–2.074, *p* < 0.001) (Table 4).

**Table 3:** Background and survival outcomes of the cardiac arrest cases (AAM implemented cases).

	G1	G2	G3	G4	*P* for trend
**Background of AAM implemented cases**					
Number of cases	18,090	28,167	41,878	75,439	
Age (y.o), mean (S.D.)	75.5 ± 14.4	76.8 ± 14.2	76.7 ± 14.3	76.1 ± 14.5	0.486
Male, n (%)	10,824 (59.8)	16,325 (58.0)	24,396 (58.3)	43,190 (57.3)	< 0.001
Presumed cardiac causes, n (%)	11,003 (60.8)	17,266 (61.3)	25,880 (61.8)	50,890 (67.5)	< 0.001
Witnessed by bystander, n (%)	7939 (43.9)	10,333 (36.7)	15,560 (37.2)	27,676 (37.2)	< 0.001
Bystander CPR, n (%)	9423(52.1)	15,143 (53.8)	24,487 (58.5)	39,319 (52.1)	< 0.001
Initial rhythm VF, n (%)	1299 (7.2)	1578 (5.6)	2621 (6.3)	4864 (6.4)	0.886
ETI, n (%)	3327 (18.3)	6340 (22.5)	5727 (13.7)	15,075 (20.0)	
SGA, n (%)	14,763 (81.7)	21,827 (77.5)	36,151 (86.3)	60,364 (80.0)	
Epinephrine, n (%)	4530 (25.0)	5968 (21.2)	11,753 (28.0)	29,779 (39.5)	< 0.001
**EMS care interval (min), mean (S.D.)**					
Call to ambulance arrives on the scene	8.3 ± 4.1	8.9 ± 4.3	8.4 ± 3.7	7.6 ± 3.3	< 0.001
Call to AAM implementation	20.2 ± 7.5	20.9 ± 8.1	18.5 ± 7.3	17.4 ± 6.1	< 0.001
Call to Epinephrine	25.2 ± 8.3	27.8 ± 8.9	24.4 ± 8.7	23.9 ± 7.9	< 0.001
Call to hospital arrival	39.1 ± 12.7	37.6 ± 12.9	34.8 ± 11.9	35.8 ± 13.1	< 0.001
**Survival outcomes of AAM performed cases**					
ROSC in the field, n (%)	1627 (9.3)	1714 (6.1)	3482 (8.3)	7372 (9.8)	< 0.001
Survival at one month, n (%)	576 (3.2)	716 (2.5)	1380 (3.3)	3104 (4.1)	< 0.001
CPC 1 or 2, n (%)	148 (0.8)	193 (0.7)	461 (1.1)	961 (1.3)	< 0.001

*CPR* cardiopulmonary resuscitation, *AAM* advanced airway management, *ETI* endotracheal intubation, *SGA* supraglottic airway placement, *EMS* emergency medical service, *ROSC* return of spontaneous circulation, *CPC* the cerebral performance category.

**Table 4:** Multivariate analysis for Survival with CPC1 or 2 (AAM performed cases).

	OR	95% CI		*P*
Age	0.968	0.968	0.974	*P* < 0.001
Male	1.132	1.001	1.281	0.048
Presumed cardiac causes	1.264	1.099	1.454	0.001
Witnessed by bystander	3.480	3.068	3.948	*P* < 0.001
Bystander CPR	1.199	1.079	1.332	0.001
initial shockable rhythm	12.54	11.10	14.16	*P* < 0.001
Epinephrine administration	0.641	0.575	0.716	*P* < 0.001
Call to ambulance arrives on the scene	0.870	0.851	0.889	*P* < 0.001
Call to hospital arrival	1.001	0.997	1.006	0.482
**AAM implementation proportion by prefecture**				
G1 (< 31.5%)	Reference			
G2 (31.5–47.4%)	1.158	0.924	1.453	0.203
G3 (47.4–59%)	1.717	1.408	2.093	*P* < 0.001
G4 (≧59%)	1.723	1.432	2.074	*P* < 0.001

*CPC* the cerebral performance category, *OR* odds ratio, *CI* confidence interval, *CPR* cardiopulmonary resuscitation, *AAM* advanced airway management.

## Discussion

This study is the first to evaluate the relationship between frequency of pre-hospital AAM approach at the prefecture level and neurological outcomes for OHCA in Japan. The study examined prefectural AAM implementation for 403,707 OHCA patients and its impact on good neurological survival at one month based on the All Japan Utstein Registry. As the AAM implementation proportion increased, the number of survivors with CPC1 or 2 also increased. In the multivariate analysis for CPC1 or 2 after adjusted confounders, high AAM implementation was associated with a better neurological outcome (CPC 1 or 2).

In previous observational studies, prehospital AAM implementation by EMS personnel has been associated with worse outcomes^8–14^. In a meta-analysis of previous studies, SGA improved ROSC rates compared to BVM (OR 1.35; 95% CI 1.11–1.63) and ETI improved ROSC rates compared to BVM (OR 1.21; 95% CI 1.01–1.44), but these airway interventions did not differ in survival to hospital discharge or neurological outcomes^25^. Izawa et al. reported that in the time dependent propensity score sequential matching for OHCA in adults, AAM was not associated with survival among patients with shockable rhythm (adjusted risk ratio 1.00, 95% CI 0.93–1.07), whereas AAM was associated with better survival among patients with non-shockable rhythm (adjusted risk ratio 1.27, 95% CI 1.20–1.35)^26^. However, some of the findings from these prior observational studies may have had biased results favoring no AAM because patients who received AAM were likely to be more severe, since ROSC could not be achieved prior to or during transportation, a state that is potentially associated with less favorable outcomes. In all observational studies assessing cardiac arrest interventions, “resuscitation time bias” (that is, the fact that patients undergoing longer resuscitation tend to receive more interventions) remains a crucial limitation^27^. This resuscitation bias in part may explain the findings of prior studies suggesting that AAM does not improve prognosis. In fact, our study also showed similar findings with negative correlation between AAM and CPC 1 or 2 outcomes at one month (Table 2). However, comparisons between the G1 to G4 groups based on the quartile of the AAM frequency across prefectures allowed us to examine the association without considering the resuscitation time bias.

Unless closely monitored, CPR quality may be interrupted when AAM is introduced into EMS systems. In this study however, at a prefecture level, the increased use of AAM did not adversely affect the prognosis of OHCA and on the contrary improved it. In the group with a high AAM implementation, AAM tended to being implemented sooner than low implementation group, but our analysis is limited without information of time required to perform AAM. Wang et al. reported that the proportion of successful first endotracheal intubation was as low as 51%^28^. Indeed, up to 20% of out-of-hospital tracheal intubation efforts are unsuccessful^29^. The low ETI success proportion may not reflect typical paramedic ETI practice, and suggests that OHCA outcomes may have been different with better paramedic ETI skills in these studies. Jabre et al., showed that physicians performed tracheal intubation for OHCA patients outside the hospital with a high success proportion, the favorable functional survival (CPC 1–2) at day 28 was 4.2% and not different than that achieved with BVM ventilation. These authors also noted that there was no significant difference between the 2 groups (BVM vs. ETI) with regards to chest compression fraction (87% in ETI group vs 86% in the BMV group; difference, − 1% [95% CI − 4% to 2%]; *P* = 0.70)^30^. Without AAM, ventilation can be performed at 30: 2, and following AAM ventilation can be performed asynchronously. Chest compressions are interrupted during AAM, but there is no need to interrupt chest compressions after AAM, which may enhance the effectiveness of CPR. Further improvement in outcomes may be expected by improving the quality of CPR, the success rate of AAM and shortening the insertion time for AAM.

### Limitations

With regards to limitations, we note that since this was a retrospective, observational study, missing data may affect our conclusions and therefore our results need to be interpreted with caution. Second, as mentioned previously there is a possibility of resuscitation time bias. Third, there are factors that cannot be investigated, such as the quality of resuscitation including CPR, the time course of the resuscitation interval, the precise location of cardiac arrest, and the electrocardiogram waveforms that require shock, but where shock was not administered. Forth, there is the issue of ‘ecological bias’. We grouped prefectures based on their use of AAM. However, there may be other unmeasured confounders that may be driving differences between groups, such geographic factors like hospital distances, system factors like ELST numbers, training for advanced life care such as AAM, the quality of AAM implementation, and financial issues required for training of advanced life care including AAM. Also regional MC committees had variations in medical protocols. Fifth, our data lack information about the in-hospital care following successful prehospital resuscitation (hemodynamic support, targeted temperature management, and coronary intervention therapy). Sixth, it was not possible to investigate the number and reasons for AAM failures and the time it took for the AAM to take place. Seventh, there is a selection bias in the AAM implementation content between regions, whether it is due to a supraglottic airway device or endotracheal intubation. Eighth, the large sample size might be leading to statistically significant differences which are not clinically meaningful. Ninth, the study has somewhat limited generalizability (single country). The applicability of the results of the study to other countries should discussed. Finally, other AAM papers have used survival to hospital discharge as an outcome, but our study used CPC1/2 at one month in survivors as the primary outcome. While this makes direct comparisons with other studies difficult, neurologically favorable survival is a more valuable patient-oriented outcome.

### Future research

There is a need for further research on pre-hospital AAM. Clinical trials wile compare AAM and non-AAM airway approaches. Studies should clearly identify the equipment used and the airway management method (e.g., whether aids were used with BVM) so that a more accurate comparison of different airway methods can be made. Resuscitation time bias remains an important issue in cardiac arrest studies, and efforts should be made to accurately determine airway intervention times to account for this concern. Studies should incorporate objective indicators of successful oxygenation and ventilation (e.g., waveform capnography, video monitoring).

## Conclusions

We found that high AAM use at the prefecture level was associated with better neurological outcomes (CPC 1 or 2) for OHCA cases in Japan. A large, well-designed research effort is needed to define the benefit from pre-hospital AAM.

## Data Availability

The datasets used and/or analyzed during the current study are available from the corresponding author on reasonable request.

## Abbreviations

OHCA: Out-of-hospital cardiac arrest
EMS: Emergency medical services
BLS: Basic life support
CPR: Cardiopulmonary resuscitation
BVM: Bag-valve-mask
SGA: Supraglottic airway placement
ETI: Endotracheal intubation
AAM: Advanced airway management
ELSTs: Emergency life-saving technicians
MC: Medical control
ILCOR: International Liaison Committee on Resuscitation
ROSC: Return of spontaneous circulation
CPC: Cerebral performance category
